# Technology, Megatrends and Work: Thoughts on the Future of Business Ethics

**DOI:** 10.1007/s10551-022-05240-9

**Published:** 2022-10-03

**Authors:** Premilla D’Cruz, Shuili Du, Ernesto Noronha, K. Praveen Parboteeah, Hannah Trittin-Ulbrich, Glen Whelan

**Affiliations:** 1 Organizational Behaviour Area, Indian Institute of Management Ahmedabad, Ahmedabad, Gujarat India; 2 Peter T. Paul College of Business and Economics, University of New Hampshire, Durham, USA; 3 College of Business and Economics, University of Wisconsin – Whitewater, Whitewater, USA; 4 Institute for Management and Organisation, Leuphana University of Lüneburg, Lüneburg, Germany; 5 Department of Organization and Human Resources, University of Quebec in Montreal, Montreal, Canada

**Keywords:** Accountability, Artificial intelligence, Big business, Corporate social responsibility, Demographic challenge, Digital technology, Ecosystem, Global production networks, Just transition, Platforms, Post-work, Religion, Robots

## Abstract

To commemorate 40 years since the founding of the Journal of Business Ethics, the editors in chief of the journal have invited the editors to provide commentaries on the future of business ethics. This essay comprises a selection of commentaries aimed at creating dialogue around the theme *Technology, Megatrends and Work*. Of all the profound changes in business, technology is perhaps the most ubiquitous. There is not a facet of our lives unaffected by internet technologies and artificial intelligence. The Journal of Business Ethics established a dedicated section that focuses on Technology and Business Ethics, yet issues related to this phenomenon run right through all the sections. Kirsten Martin, editor of the Technology and Business Ethics section, joins our interim social media editor, Hannah Trittin-UIbrich, to advance a human-centric approach to the development and application of digital technologies that places Business Ethics at centre of the analysis. For Shuili Du, technology is the defining condition for a new era of Corporate Social Responsibility—CSR 3.0—which she defines as “a company’s socially responsible strategies and practices that deal with key ethical and socio-technical issues associated with AI and related technologies on the one hand and leverage the power of AI and related technologies to tackle social and environmental problems on the other hand.” It is not just technologies that are a determining feature of our lives but technology companies, an argument made by Glen Whelan as he examines Big Business and the need for a Big Business Ethics as we try to understand the impact of Big Tech on our post-work world. Indeed, as noted by Ernesto Noronha and Premilla D’Cruz, megatrends in addition to advancement in technologies, namely globalization, the greening of economies, and changes in demographics and migration, are shaping the future for workers in ways previously unimaginable. Contributing to this important debate, Praveen Parboteeah considers the influence of another longstanding but oft overlooked megatrend, the role of religion in the workplace. Given the enormity of the influence of technology and other megatrends in our world, it is not surprising that this essay introduces ground-breaking ideas that speak to the future of business ethics research.

## Towards a Human-Centred View on Digital Technologies

**Hannah Trittin-Ulbrich and Kirsten Martin**

### Introduction

The ethical concerns emerging from the proliferation of digital technologies have attracted attention from not only the popular press, but also a growing number of scholars across disciplines. This is not surprising, given that technology has always been met with ethical and critical examination. Arguably, the printing press and pony express were both viewed as disruptive innovations with value-laden design decisions and ethical implications. The steam engine was seen as an abomination against the gods by combining water and fire. And, the critical scholarly examination of technologies has included bicycles, plastics, seatbelts, bridges, among many other technologies (e.g. Winner, 1980). Even within our more current innovations, the ethical examination of digital technologies has advanced within engineering and philosophy for decades (e.g. Johnson, 1985).

However, we, that is, business ethicists, should not leave the interrogation of ethical concerns of digital technologies to ethicists of other disciplines (i.e. AI/artificial intelligence ethics scholars). In this essay, we argue that our discipline is uniquely equipped to interrogate the ethical implications of digital technologies for business and society. We use the term digital technologies to mean information and communications technologies that rely on the latest data analytics techniques to include a range of technologies, e.g. artificial intelligence (AI), social media, platforms, facial recognition or blockchain. Digital ethics, defined here as current efforts to discern the ethics of, and corporate responsibilities for, digital technologies, provide important counter-perspectives to the tech-hype that is fueled by the “Internet-industrial” complex (Flyverbom et al., 2019), that is, those private, public and other actors involved in the development and governance of the Internet and digital technologies.

With this essay, we hope to inspire future business ethics research to further interrogate what constitutes a human-centred approach to the development and application of digital technologies in the business context. We propose that a critical, human-centred approach implies that digital technologies should be developed and adopted in the interest, and to the benefit, of those individuals who are affected by them. To that end, importantly, we must avoid falling into the trap of (unintentionally) subscribing to imperative arguments regarding the inscrutability, efficiency and profitability of digital technologies. Let us explain.

### What Business Ethicists Should Avoid When Dealing with Digital Technologies

First, digital technologies are often sold with the false claim of *inscrutability*. Artificial intelligence, in particular, has often been debated as working in a “mysterious”, autonomous way. In view of such autonomous decision making of artificial intelligence, firms and their representatives claim that they can no longer be held accountable for the impact that such technology produces. Automated decision making is also suggested as creating fairer, more objective outcomes than human beings. And yet, if an algorithm is found to create wrongful or even harmful outcomes, firms tend to displace responsibility towards the autonomous artificial agent: “It's not us, it's them”. However, digital technologies designed to be inscrutable are more about corporate power than any design requirement (Kroll, 2018; Pasquale, 2015). Engineers have developed ways to test and report ethical issues of AI and even machine learning—that is, the design decision to make a programme inscrutable is a decision and should not be taken as a given (Martin, 2019).

This brings us to the second fallacy of digital technologies: digital technologies also (falsely) promise *efficiency* and the hyper-rationalization of firm activities. Goodbye slow, flawed human decision making, welcome rational and efficient automated decision making! Brave new digital world. Arguably, such narration relies on two flawed assumptions: (1) a limited, economic theory of the firm that conceives of the business firm as a purely economic actor, whose only goal must be the enlargement of the shareholder value through continuous enhancement of the firm’s efficiency; and (2) a view of technology development as neutral and objective, and devoid of value-laden decisions made throughout the design and development process (Martin, 2022).

Finally, digital technologies are also falsely hailed as suitable means to increase corporate *profitability*. We are told that digital technologies allow businesses to “move fast and break things” (as famously proclaimed as the key to success of the social networking platform Facebook by CEO [Chief Executive Officer] Mark Zuckerberg), that is, to disrupt existing markets and industries, allowing them to quickly enlarge their value and attractiveness for venture capital. In this sense, the efficiency of digital technologies is promised to directly result in a firm’s increase in profitability.

Questioning these three common assumptions of inscrutability, efficiency and profitability is not enough in pursuing a more critical, human-centred approach to digital technologies. We therefore now turn to identifying three unique avenues of scholarship on the ethics of digital technologies where business ethics research has a unique grounding and perspective that allow for a human-centred view on digital technologies.

### Where Business Ethics Should Engage

#### Who Should be Held Accountable for the Impact of Digital Technologies?

First, in order to move business ethics research forward, we must avoid falling into the trap of thinking that businesses cannot be held accountable for the moral implications of the digital technologies they use or produce. Arguably, decision making augmented with AI often takes place behind closed (corporate) doors, hiding from public scrutiny and oversight. However, business ethics research is essentially the study of accountability: that is, the field questions who is responsible for an action or outcome under which premises, as well as providing reason as to *why* firms are responsible for their decisions and their impact on society.

Mistakenly assuming that digital technologies provide more efficient, accurate decisions, and are outside the realm of any critical examination or moral evaluation leads scholars to incorrectly see the development of digital technologies as being deterministic and outside their scope. Judging technologies on efficiency and treating digital technologies as inscrutable products also shields corporations from being held accountable for the value-laden decisions made in the design, development and deployment of algorithms. At the other end of the spectrum, pretending that digital technologies are only as ethical as how society uses them—as if the design decisions have no bearing on the moral implications of their use—allows firms who design and develop digital technologies to avoid the sharp gaze of critical theorists who wish to hold them responsible for their decisions.

To lift the “veil of the technological imperative” (Martin, 2022) and critically examine the moral implications of the design, development and use decisions around digital technologies, business ethicists should raise questions in regard to who can be held accountable for how digital technologies impact business and society, as well as to why this is so. In particular, we must further interrogate the design and development process of digital technologies, and must ask how we can hold those actors, including information technology experts or software developers, accountable for their decisions in this process. Business ethics scholars should follow others who acknowledge that digital technologies have biases that are value-laden (Friedman & Nissenbaum, 1996; Johnson, 2004) or have political dimensions (Winner, 1980), while also identifying how individuals, corporations and society can control that same technology.

Digital technologies have value-laden biases along several dimensions which can serve as avenues for future normative work within business ethics (Martin, 2022). In particular, digital technologies are biased towards and designed for a preferred set of actions that create (or destroy) value for stakeholders, uphold (or violate) ethical principles and reinforce (or undermine) stakeholder rights and dignity. In this way, digital technologies can be seen as embodying corporate policy or the norms and rules of the organization which are then enacted into decisions. As such, business ethics should expand the ethical evaluation of digital technologies to include the value-laden decisions made around the outcome of digital technologies, the criteria for whether a technology *works*, the choice of data used as well as the assumptions made in the development of these technologies.

#### How do we Theorize the Goal of the Firm in Relation to Digital Technologies?

Business ethics research is also uniquely equipped to counter the imperative arguments regarding the efficiency and profitability of digital technologies. Our field has a longstanding tradition of questioning one-sided and purely functionalist constructions of the goal of the business firm and, therefore, should naturally respond with caution to idealizations of the automated firm.

In business ethics, we argue (regularly) that the focus on shareholder value is mistakenly socially constructed as the only goal a firm should pursue. Similarly, the idea of efficiency as the only goal of digital technologies is socially constructed and inherently value based. Indeed, efficiency is usually constructed to serve only specific sets of actors—the firm and its shareholders—without consideration of other actors that have a stake in the firm’s activities and involvement with digital technologies. For example, for whom is the hiring AI programme efficient? How are the goals of the firm served if an AI programme to read resumes or assess interviews consistently makes mistakes, but does so “efficiently”? Business ethicists aiming to advance our field in the study of the ethics of digital technologies should carefully interrogate and expose the connection between the development and use of digital technologies that can and should be aligned with a thicker conception of the goal of the firm. Much of the current work on the ethics of digital technologies relies on a thin, shareholder wealth maximizing view of the firm.

The case of social media content moderation algorithms—designed to quickly promote *lawful but awful* material in order to increase user engagement—exemplifies how digital technologies may serve a specific goal while being destructive to firm value from the perspective of society at large. Within the field of AI ethics, Thomas & Uminsky (2020) call for multifaceted outcomes for measuring the effectiveness of data analytics programmes and even the danger of allowing an analytics programme’s outcome variable, which is being optimized, to dominate the decision making of the firm. Social media’s fixation on user engagement, to the detriment of all other measures, exemplifies this danger.

Broadening notions of the goal of the firm also requires broadening conceptions of the value of digital technologies. Many proponents of the digital economy try hard to promote a dichotomous concept of digital technologies as only productive if unconstrained by governmental regulation around fairness. To contribute to a more humane approach to digital technologies, business ethics scholars should not approach fairness and efficiency as opposing, but rather as complementary, goals in the application of digital technologies. We must critically interrogate when, and under which conditions, businesses can achieve both a fair and productive application of digital technologies, and thus, also contribute to fairer and productive businesses. We must ask, how can a corporation live up to its corporate digital responsibility? Moreover, how do societal demands shift and change in regard to corporate digital responsibilities, and what implications emerge from changing and evolving societal expectations regarding fair and responsible business conduct for the application of digital technologies? Who are the stakeholders of digital technologies, and how should technologies be designed and developed with these stakeholders in mind?

Finally, to contribute to a humanistic approach to digital technologies, business ethicists should further interrogate the question who the stakeholders of digital technologies are and how technologies should be designed and developed with these stakeholders in mind. In the past, business ethics research has provided much insight into how and why businesses should respect the interests and rights of various stakeholders. Following this line of reasoning, business ethics scholars should interrogate how the business application of digital technologies enhances both the firm's performance and contributes to serving the interests of all stakeholders.

In business ethics, stakeholders are generally considered those individuals that are affected by or can be influenced by a business decision or action. The general assumption here is that stakeholders engage knowingly and often voluntarily with businesses and have some sort of formal relationship with the firm, for example, by being their customers, employees, suppliers (Freeman, 1984). Normally, to categorize stakeholders and prioritize their interests, we distinguish primary and secondary stakeholders, and group them into a “manageable” stakeholder community that a business regularly engages with. Stakeholders are voluntary, we assume, and in a relationship with the firm.

However, with digital technologies, our current approach as to who constitutes a stakeholder is challenged. Not only are decisions augmented with AI hidden from market governance or public oversight, but digital technologies impact actors that have no formal relationship with the firm and are not voluntary. Social media content moderation algorithms impact not only the advertisers, which are customers and serve as revenue sources, but also users and even individuals and groups not on social media. The recommendation of hate groups by algorithms or violence against dissidents impacts not only the users of social media, but the targets of these violent groups who are not on social media. The original definition of a firm’s stakeholder—those who are influenced by or influence the firm (Freeman, 1984)—is a closer approximation to the issues faced today with digital technologies. Such a definition does not require stakeholders to be voluntary, nor in an immediate relationship with the firm. It is therefore crucial that ethicists begin by reviewing who the stakeholders of digital technologies are and whose voices should be considered when developing them.

Based on the original definition of stakeholder (Freeman, 1984), we see three types of unaware and often overlooked stakeholders, where business ethics research could leverage our theories to understand how firms should manage these stakeholder relationships. First, stakeholder groups of digital technologies may include, for example, “unaware” stakeholders who are being impacted by a digital technology but are not aware of the digital technology being used. Unaware stakeholders include individuals whose job application, social networking or dating site pictures on the Internet are used to train face recognition technology. Similarly, this type of stakeholders includes those confronted with the results of automated decision making without realizing that such technology is applied. Since some technologies may crawl and detect data points in the global Internet, this makes any Internet user potentially a part of this stakeholder group—reducing the very idea of a more or less stable and controllable “stakeholder community” to absurdum.

Second, while arguably, many relations businesses traditionally hold with their stakeholders are slanted in terms of distribution of power or information, digital technologies create new “unequal” stakeholders that interact with businesses in the form of unequal relations. These include “gig” or platform economy workers that often work at the whim of an algorithm, gaining work assignments and being evaluated through opaque forms of algorithmic management and control. Similarly, platform users, including social media users, have limited insights into what algorithmic decision has led to certain content being shown to them, while other remains hidden. Even businesses may find it hard to gain insights into why e-commerce platforms decide to show their products only to certain customers, reducing even large business conglomerates to an “unequal” business partner to these platform titans.

Third and finally, digital technologies also create new “invisible” stakeholders, that is stakeholders that are invisible to the digital economy business models. Invisible from the public eye, and often hidden in plain sight from scholarly inquiry, masses of poorly paid independent contractors from the global south “curate” the content that is published on social media platforms by reviewing and deleting masses of disturbing and often downright illegal data. Other stakeholder groups of this category also include those factory workers working in the delivery centrs of large e-commerce retailers.

These stakeholders are, within our current parlance, legitimate but marginalized. Future business ethics research concerned with digital technologies should interrogate how businesses should have an obligation for the impact their technologies have on these and other stakeholders. How should digital technologies be designed to give unaware and/or silent stakeholders a voice? Which role can artificial intelligence play in creating new forms of automated accountability? How can the rights of those being unaware of their status as being a stakeholder be upheld? How can more transparent and fair working conditions be ensured in platforms, and how can workers’ dignity and rights be secured in fully automated work arrangements that lack governmental regulation and public oversight? Providing answers to these questions will contribute to a more critical, human-centred approach to digital technologies.

### Conclusion

With this essay, we hope to inspire future business ethics research to further interrogate what constitutes a human-centred development and application of digital technologies in the business context. The proliferation of digital technologies promises great human advancement, while also raising questions regarding the ethical and responsible development and application of these technologies by businesses. We welcome the growing number of business ethicists paying attention to the critical, problematic and “dark” implications of these technologies for business and society (e.g. Trittin-Ulbrich et al., 2021). We have outlined imperative arguments regarding the inscrutability, efficiency and profitability of these technologies and we have outlined three areas of growth for future research through which business ethics scholars can contribute to a more critical and human-centred approach to digital technologies. We are looking forward to seeing their efforts!

## Reimagine Corporate Social Responsibility in the Age of Artificial Intelligence

**Shuili Du**

### Artificial Intelligence and its Double-Edged Effects

As artificial intelligence (AI) increasingly permeates the business world and modern society, companies need to rethink and broaden the scope of their corporate social responsibility strategies and initiatives to deal with key ethical and socio-technical issues triggered by AI and related technologies. Defined as “the ability of machines to carry out tasks by displaying intelligent, human-like behavior” (e.g. machine learning, computer vision, speech recognition and natural language processing: Russell & Norvig, 2016), AI is transforming our economy. The global AI market size is forecast to grow from $58.3 billion in 2021 to $309.6 billion by 2026, at a compound annual growth rate of 39.7% (Markets & Markets, 2021). AI technologies are being deployed in diverse sectors, ranging from finance, health care and transportation, to national security, criminal justice and smart cities, augmenting human capabilities in significant ways and making a profound impact on the world. However, AI is a mixed blessing. On the one hand, it promises scientific breakthroughs and advancement of humanity with its superior processing speed, limitless recall and self-improving learning ability. On the other hand, it is fraught with a host of unprecedented ethical and socio-technical challenges, such as AI algorithmic biases, machine ethics, data privacy, job replacement by AI and exacerbated digital inequity.

AI follows the trajectory of exponential growth, and it seems that our society is marching inexorably towards artificial superintelligence—the point of singularity—when AI systems will be self-aware and outperform humans in nearly all areas (Bostrom, 2014). Super-intelligent AI will be capable of complex goal setting and can engage in scientific discovery and artistic creativity (Tegmark, 2017). Such systems hold enormous promise in transforming every aspect of our society for the better by, for example, repairing damage done to the natural world and eradicating poverty and diseases. At the same time, when machine intelligence eclipses human intelligence, technological growth becomes uncontrollable and irreversible, resulting in unforeseeable changes to human civilization. AI is humanity’s biggest existential threat, as Elon Musk famously stated.

The future as increasingly mediated by AI is both fascinating and terrifying. Corporate social responsibility (CSR) scholars can play a big role in shaping the short-term and long-term future of ethical and socially responsible AI. To embrace the power of AI while minimizing its downsides, companies should reimagine their CSR strategies and practices to turn the unique social challenges of AI into business opportunities. In the short term, businesses need to tackle an array of ethical and socio-technical issues surrounding AI nowadays, including AI biases, machine ethics, data privacy, cybersecurity, individual autonomy, job replacement by AI, digital inequity and so on. In the long term, businesses and society face the ultimate challenge of ensuring that super-intelligent AI will act in the best interests of humanity, which is no easy feat since superintelligence could far surpass human intelligence and be unstoppably powerful. Accordingly, the dialogue on how to make super-intelligent AI human-friendly needs to take place now and be much more inclusive, engaging not just computer programmers, mathematicians and AI scientists, but also business scholars, philosophers, sociologists and ethicists. With its powerful, unprecedented capabilities and its unique ethical and socio-technical challenges, AI raises many new and important research questions for CSR scholars, recasting and expanding the substantive domain of CSR research. Conversely, the field of CSR, with its unique focus on the intersection of business and society and its rich, accumulated insights from a large body of literature, has much to offer to the vital dialogue of how to develop ethical and socially responsible AI.

### Evolution of CSR: Looking Back and Looking Forward

The field of CSR has undergone several evolutions in reaction to the big trends in the macro-environment, and currently the exponential growth of AI and its related technologies (e.g. big data, machine learning, Internet-of-Things) are prompting another round of CSR evolution (see Table 1). From Milton Friedman’s famous 1970 article “The Social Responsibility of Business is to Increase Its Profit” to the 2019 Business Roundtable’s new Statement on the Purpose of a Corporation, affirming businesses’ fundamental commitment to all stakeholders, the field of CSR has come a long way. Under CSR 1.0, shareholder primacy was the norm. Corporate scandals like the Exxon Valdez oil spill in 1989 and the Nike sweatshop controversy in the 1990s evoked public outcry and pushed companies to think about the moral imperative of their businesses. Companies started to engage in CSR initiatives. Yet the underlying premise of CSR 1.0 is that business and social interests are contradictory, and the prevailing approach to CSR is reactive, ad hoc, short-term oriented and disconnected from business strategy. CSR was considered a cost of doing business and merely functioned as a public relations tactic to garner community goodwill.

**Table 1:** Evolution of CSR

	CSR 1.0	CSR 2.0	CSR 3.0
Timeline	Prior to 2000	2000—2020	2020—forward
Fundamental premises	Business and social interests are contradictory; **CSR as cost of business**	Business and social interests are interdependent; **CSR as a source of competitive advantage**	Business, society and technology as interdependent; **CSR as a necessary means to shape the future of socially responsible AI**
Defining characteristics of CSR practices	Companies engage in CSR due to moral considerations or stakeholder pressure; CSR practices are reactive, ad hoc, short-term-oriented and disconnected from business strategy; CSR functions as a public relations tactic to garner community goodwill	Companies engage in CSR because it is not only the “right thing”, but also the “smart thing” to do; CSR practices are proactive, systematic, long-term-oriented and aligned with business strategy; CSR functions as an integral part of business strategy and creates joint social and business value	A broadened conceptualization of CSR by encompassing an element of technological social responsibility; companies use CSR principles to address the ethical and socio-technical issues of AI and related technologies; companies leverage the power of AI to tackle complex social and environmental problems
Stream of literature	Friedman (1970)	Sen & Bhattacharya (2001), Porter & Kramer (2006)	Du & Xie (2021), Islam (2021)
Triggering events for the evolution of CSR	Exxon Valdez oil spill in 1989; Nike sweatshop scandal in 1990s; Greenpeace protests, in 1995, of Shell Oil’s decision to sink the Brent Spar, an obsolete oil rig, at sea	UN Global Compact launched in 2000, encouraging businesses to adopt socially responsible policies; Enron scandal in 2001; 2007 Great Recession; public awareness of climate change; increasing stakeholder expectations of businesses	Facebook–Cambridge Analytica data breach in 2018; growing disillusion with the exploitative nature of smart technologies; EU (European Union) General Data Protection Regulation (GDPR) in 2018; Apple rolled out new privacy protection measures in 2021

Starting in the early 2000s, the confluence of several trends—the launch of the UN (United Nations) Global Compact, the Enron scandal, public awareness of climate change and increasing stakeholder expectations for businesses—ushered in CSR 2.0. In this era, companies engage in CSR not only because of the moral imperative, but also the business imperative: business and social interests are no longer considered a zero-sum game but interdependent and complementary. The prevailing approach to CSR is increasingly proactive, systematic, long-term oriented and aligned with business strategy. CSR has moved from the periphery to the core of business and becomes an integral part of business strategy and a source of competitive advantage (Porter & Kramer, 2006). More and more companies release annual CSR reports, communicating their CSR strategies and key performance indicators in various social and environmental domains. Academic research on CSR flourishes, with numerous studies documenting the business benefits of CSR as well as contingent factors that could accentuate or diminish these benefits.

From 2020 onwards, the exponential growth of AI and its plethora of ethical and socio-technical issues have prompted the need for business managers and academic scholars to revamp the notion of socially responsible business strategies and practices, ushering in CSR 3.0. The infamous Facebook–Cambridge Analytica scandal serves as a wake-up call to both companies and consumers about protecting their digital data privacy; documentaries on AI such as *The Social Dilemma* and *Code Bias* reveal many harmful effects of AI on individual and societal well-being. As companies navigate the uncharted waters of AI-mediated business landscape, they urgently need to update their model of CSR to proactively address the complexities and challenges associated with the increasing deployment of AI in their businesses. We define CSR 3.0 as “a company’s socially responsible strategies and practices that deal with key ethical and socio-technical issues associated with AI and related technologies, on the one hand, and leverage the power of AI and related technologies to tackle social and environmental problems, on the other”. The fundamental premise of CSR 3.0 is that AI technologies, businesses and society are intricately interdependent, with CSR being a necessary and essential means to shape the future of socially responsible AI. CSR 3.0 encompasses a technological component—technological social responsibility—in its conceptualization, and emphasizes a systematic, long-term-oriented approach in companies’ development and utilization of AI and related technologies. In other words, ethical and social considerations of technologies should be embedded in companies’ core business strategy, not as an add-on or a public relations tactic.

We call for ground-breaking and boundary-spanning CSR research to examine (1) how AI technologies can incorporate ethical and socially beneficial features and the consequent effects of such features on AI’s social legitimacy and marketplace performance, (2) how companies can develop a strategic approach to AI-related CSR to cultivate their competitive advantage and create shared social and business value in an AI-mediated economy, and (3) how an ecosystem of businesses, government institutions, non-profits, stakeholder advocacy groups and others could collectively promote the long-term symbiotic coexistence of machine and human intelligence.

### CSR 3.0 Research Directions: Enhancing the Ethical and Social Aspects of AI

**Fig. 1:**
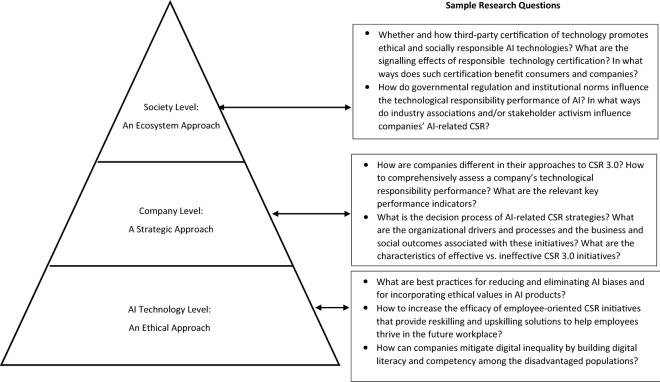
Three Levels of CSR Research in the Age of AI

The double-edged nature of AI brings unprecedented opportunities and challenges for companies in their efforts to be good corporate citizens. Below we provide an overview of CSR 3.0 research directions at the AI technology level, the company level and the society level (Fig. 1). This section is not intended to be exhaustive, but rather to kickstart dialogues on future-oriented CSR research that addresses the complexities and opportunities of AI and promotes human flourishing in a technology-mediated future.

#### AI Technology Level: An Ethical Approach

One promising avenue of future CSR research is to examine how AI technologies could be more ethical and socially beneficial. Due to the numerous ethical challenges and negative social ramifications associated with AI, society and stakeholders often view AI technologies as untrustworthy, scary and even malicious (Cave et al., 2019), which hinders AI’s social legitimacy and stakeholders’ adoption of such technologies. Undoing such scepticism and mistrust will require companies to demonstrate that AI technologies provide real social benefits and have embedded ethical values.

In the consumer domain, AI algorithmic biases and machine ethics are two noteworthy issues. Research has shown that AI algorithms show bias related to gender, race, ethnicity, geography and other socio-economic variables (e.g. education, income, zip codes), and in particular, disadvantaged consumers are likely to be negatively affected when companies incorporate AI in their products and services (Zou & Schiebinger, 2018). Machine ethics is another pressing issue as more and more products (e.g. self-driving cars, robo-advisors, virtual nurse assistants) make autonomous or semi-autonomous decisions with real-world consequences. It is critical to properly integrate ethical principles in AI products as well as to ensure the requisite alignment of ethical values between the product and the user. In addition, other AI issues in the consumer domain include digital data privacy, cybersecurity and profit-maximizing algorithms that have detrimental effects on consumer mental health and well-being. Research could examine and promulgate best practices for embedding ethical and socially beneficial features in AI that promote consumer and societal well-being.

In the employee domain, AI will have a profound impact on the future of work. On the one hand, AI can liberate humans from undesirable tasks (e.g. strenuous and dangerous physical work, tedious and repetitive tasks), enhance workplace safety and make our jobs more fulfilling. On the other hand, automation will eliminate many jobs, resulting in large-scale unemployment or underemployment. Jobs not only are a means for earning an income, but also fulfil important psychosocial needs of individuals, such as self-worth, achievement, social status, respect, social belonging and so on. Hence the impact of AI on employment will have far-reaching societal and political implications. It is important to examine employee-oriented CSR initiatives that provide reskilling and upskilling solutions and help employees acquire relevant skills to work in an increasingly AI-mediated workplace.

In the community domain, exacerbated digital inequity—disparities in access to and use of AI and related technologies (e.g. the Internet, mobile apps, social media, smart devices)—is a pressing issue. Research has shown that AI and related digital technologies tend to increase existing social inequities in terms of gender, race, ethnicity, age, geographic location and socio-economic status (Lutz, 2019). Digital inequities manifest in three levels: inequities in access to digital devices and technologies, in skills and uses of digital technologies, and in offline benefits and harms from digital technologies (Lutz, 2019). With the centrality of AI and related technologies in the modern world, digital inequities profoundly impact life outcomes, ranging from relationship quality and academic performance to career opportunities and entrepreneurship. Further, disadvantaged populations are also more vulnerable to the harms of digital technologies, such as fraud, identity theft and digital surveillance. Thus, an important research direction is to examine community-oriented CSR initiatives that help disadvantaged population groups not only gain access to digital technologies, but also build digital competence and digital engagement.

In the diversity and inclusion domain, the field of AI has a diversity crisis, with women and minorities severely underrepresented (Howard & Isbell, 2020). A diverse workforce in the AI field could produce a broader, more heterogeneous knowledge base and a better decision-making process by exploring a wider range of perspectives. In turn, this would help firms develop more inclusive and socially responsible AI products by, for example, mitigating AI algorithmic bias and catering to the diverse needs of consumer segments. Companies can adopt various initiatives to promote diversity, such as mentoring talented women and minorities in STEM (science, technology, engineering and mathematics) fields, implementing more inclusive hiring and promotion practices and building cross-disciplinary taskforces with expertise in not only computer science and engineering, but also literature, arts, philosophy and social sciences. Future research can shed light on the effectiveness of these different approaches to addressing the diversity crisis in the field of AI.

#### Company Level: A Strategic Approach

A company’s strategic approach to CSR 3.0 should not be one size fits all. Given limited resources and differential corporate capabilities, companies should identify and prioritize relevant AI issues and design CSR initiatives aligned with their core capabilities to create more social and business value. In terms of issue selection, managers should identify AI issues that matter the most to their key stakeholders and that have a good fit with their corporate capabilities and brand positioning. To illustrate, a fintech company or a medical AI company is well advised to tackle AI algorithmic bias. because it directly affects the quality of AI products and consumer well-being, whereas an automobile manufacturer using AI and robots in its factories should focus on employee-oriented training and career counselling to help its employees expand their knowledge and skills, enabling them to work alongside AI or tap into newly created job categories.

Moreover, in addition to addressing the downsides of AI and making it more virtuous, forward-looking companies should also harness the power of AI to turbo-charge their current CSR initiatives. AI can make companies’ CSR initiatives far more effective due to its unparalleled ability to process massive unstructured data and uncover novel solutions to pressing social and environmental issues. AI and related technologies hold great promise in reducing overproduction, inventing climate-friendly materials, supporting causes in environment, health, education and disaster relief. For example, Microsoft has a series of initiatives leveraging AI to create social change and drive innovation, including AI for the Earth, AI for health, AI for accessibility and AI for humanitarian action. Stonyfield Farm establishes an AI-based software platform to help farmers improve soil health and sequester more carbon.

After issue selection, managers need to decide on the executional elements of their CSR initiative. For example, to tackle AI algorithmic bias, a company could take a technical approach by improving the training dataset and modifying the algorithm; alternatively, it could focus on the consumer experience side by providing more transparency and consumer education regarding its AI algorithm. Other executional elements of a CSR 3.0 initiative include whether to adopt a top-down or bottom-up approach, whether to establish a cross-sector partnership to leverage the expertise of a third-party organization and how to communicate the initiative to the public and engage stakeholders to co-create value.

Future research should examine how companies can design effective CSR strategies in the age of AI. It would be worthwhile to establish a typology of different types of CSR 3.0 initiatives and propose frameworks and metrics to comprehensively assess a company’s technological responsibility performance. We call for more research on the decision process of AI-related CSR strategies and characteristics of effective vs. ineffective initiatives. It is also important to understand the organizational drivers and processes and the business and social outcomes associated with these initiatives, and to identify the unique characteristics of CSR 3.0.

#### Society Level: An Ecosystem Approach

The evolution of AI technology takes place within the broader social and institutional system, consisting of government regulations, institutional norms, non-profits, advocacy groups and the competitive dynamics in the marketplace. These social, cultural and economic forces interact with technological capabilities in the business sector to influence the future trajectory of AI. Thus, CSR research should also incorporate a macro-level perspective.

The future marketplace is likely to have a constellation of AI products and services (e.g. digital virtual assistants, domestic robots, self-driving cars, medical robots, to name but a few), each with varying configurations of cognitive/computational intelligence and moral and social intelligence. There is a need for third-party certification organizations to provide ratings for AI products on cognitive, ethical and social dimensions and/or to certify AI products as ethically or socially beneficial if they meet certain standards. Such a certification process will reduce information asymmetry, enabling consumers to make informed decisions when purchasing AI products and allowing technologically responsible companies to reap coveted benefits such as enhanced brand image and price premium for their AI products. Future research can examine key dimensions of technological responsibility performance and relevant metrics (e.g. algorithmic biases, explainability, inclusive design, privacy protection and cybersecurity), as well as the signalling effect of technological responsibility certification and the benefits of such certification to consumers and companies.

Future society is likely to have a constellation of organizations, each playing a unique role in addressing the ethical and social challenges related to AI and promoting the long-term symbiotic relationship between machine and human intelligence. There is a need for future research to inform government and public policy makers, industry associations, non-profits and various stakeholders on how to tackle the challenges facing us. Countries differ in essential aspects such as governmental approach to AI regulation, institutional norms and cultural values, levels of stakeholder activism and developmental stages of AI technologies, all of which affect businesses’ approach to AI and their overall CSR performance. We call for future research to shed light on the impact of these macro-level dynamics on companies’ technological responsibility performance and the associated social and business outcomes.

### Conclusion

To shape the future of ethical and socially responsible AI and promote human flourishing in a future mediated by AI and related technologies, companies need to reimagine their CSR strategies and practices. There is fertile ground for novel, ground-breaking and impactful research on CSR in the age of AI.

## Big Business Ethics and a Post-Work World

**Glen Whelan**

### Introduction

This commentary proposes that, given the continued growth of the world’s largest corporations, and of Big Tech in particular, there is a clear need to develop a “Big Business Ethics” literature. Among other things, this literature would focus on analysing the ways in which market-dominating firms are not just system-takers that are influenced by extant moral and social norms, but system-makers that can transform them. To illustrate, the commentary suggests that, given their clear interest in developing labour displacing technologies, and the key role they play in enabling remote work practices, Big Tech firms may be contributing to the gradual emergence of a post-work world in which business in general, and hence, business ethics too, will cease to be of importance.

### The Need for Big Business Ethics

Evidence suggests that market concentration is growing in many industries worldwide. A major factor is that leading corporations have proven adept at using technology to reduce labour costs and increase margins (e.g. Andrews et al., 2016). While this trend towards “winner takes all/most” markets is found in many sectors, it is perhaps most clearly evidenced by the emergence of Big Tech: i.e. Alphabet (which includes Android, Google and YouTube), Apple, Amazon, Meta (which includes Facebook and Instagram) and Microsoft. These five corporations control technologies that many people consistently use in their daily lives. Consequently, the market valuation of these corporations—which in February 2020 was equal to 19% of all firms listed on the S&P500 (Standard and Poor's 500) index (Tambe et al., 2020: 2)—has grown massively over recent years.

This growth of Big Tech is fuelled by numerous factors, including behaviours that many would consider anti-competitive. Nevertheless, Big Tech’s successes have been informed by more innocent considerations, too, such as network effects, their digitally enabled scaling capacities and their provision of web hosting and machine learning services to smaller entities. As Big Tech has become ever-more prominent, many have sought to curtail their collective influence. There are various liberal and democratic reasons for wanting to do this: most of which boil down to the belief that centralized and unelected corporate elites should not be allowed to dominate markets, manipulate populations, or exert excessive control over (elected) politicians. Such negative sentiment may be growing, but there is little reason to believe that a widespread popular revolt will overthrow Big Tech in the immediate future. Moreover, concrete efforts to cut Big Tech “down to size” have hitherto inflicted flesh wounds at best. The European Union (EU), for example, has a long track record of imposing impressive individual fines, numbering in the billions of Euros, on Big Tech. But such massive fines are yet to stop Big Tech from earning ever-larger profits.

If prior decades are anything to go by, then, Big Tech and other big corporations (e.g. those in the top 5% of an industry) will continue to capture an increasing share of markets in the decades to come (e.g. Andrews et al., 2016). No doubt, predictions are difficult to make (especially about the future). But the fact that these corporations can reinvest their profits to further increase their profits—sometimes in industries that lie far afield from that in which they initially created their wealth (Whelan, 2021: 43–67)—suggests that the already big could continue to increase in size, or that, if they are superseded, it will be by even bigger corporate foes.

The time thus seems right to develop a literature on what can be termed, with no imagination whatsoever, Big Business Ethics. Indeed—when it is noted that the “small business ethics” literature has been growing for some time (Moore & Spence, 2006), and that the international relations field (which has a comparable mix of descriptive, normative and organizational concerns to business ethics) has long studied the world’s most powerful states (e.g. Mearsheimer, 2001), the need to develop a literature that is specifically focused on phenomena that are (uniquely) related to the world’s largest corporations appears long overdue.

To suggest that business ethics needs to develop a literature that is specifically focused on the world’s biggest corporations is not to suggest that there are no prior works of relevance. There very clearly are. Extant conceptual works on the form and responsibilities of corporations, for example, which are themselves built upon longstanding traditions in moral and political philosophy, help to identify, and to make sense of, moral considerations of relevance to the world’s corporate giants (e.g. Donaldson, 1982).

But what is arguably unique about the likes of Big Tech is that they have the potential to be system-makers and not just system-takers. In terms of ethics and morality, this means that the world’s biggest corporations are not simply subject to extant moral concepts and norms, but agents that can transform or redirect them. In this fashion, Big Business Ethics can be conceived as focusing on how the world’s largest corporations contribute to morally important and interesting transformations of broad, societal-level relevance. Moreover, Big Business Ethics can be understood as relating to transformations that risk going unseen because they are ubiquitous. Thus—and while Big Business Ethics obviously focuses on corporations that are unique in terms of their size and power—it does not focus on social transformations that are unique, or some sort of outlier. Rather, it focuses on transformations that are widespread and commonplace, and that are, in some sense at least, era defining. In other words, Big Business Ethics can be understood as focusing on the wood (or the forest) more than it does the trees.

The sorts of general phenomena that Big Business Ethics directs attention to include Big Tech’s transformative impact upon how individuals understand and respect their own, and other people’s, personal privacy; how people and organizations understand, and can be held to (moral) account for, their historical acts; and how people understand, and can participate in, the governance of organizational, governmental and social affairs. But Big Business Ethics directs attention to other less acknowledged developments too. One such development is the role that the world’s largest corporations are currently playing in the possible emergence of a post-work world. Thus, it is in using the remainder of this commentary to briefly discuss this possibility, that I hope to illustrate—or, somewhat more modestly, to begin to suggest—how the lens of Big Business Ethics can help to shine a light on, and begin to theorize, changes in the ethical (and/or moral) fabric of daily life.

### The Possibility of a Post-Work World

As previously noted, the Western world’s most productive firms have, since the turn of the century, captured an increasing share of markets across industries (e.g. Andrews et al., 2016). The reason why relates to leading corporations enjoying relatively high productivity rates and margins, and to their out-muscling or out-thinking smaller, less capable firms. In possessing the capacity to satisfy the preferences of huge audiences with very low marginal production costs, Big Tech can once again be understood as exemplary of such developments. More specifically, Big Tech can be used to show that very large profits can be won by corporations that (1) (massively) reduce the labour (and overall) costs associated with their own activities, and that (2) create products and services that (massively) reduce the labour (and overall) costs of their user populations. When these two points are noted, the possibility that (3) Big Tech could also play a catalytic role in the development of a post-work world comes to the fore.

To get a better sense of all this, it helps to refer to Amazon, and to note that their (in)famous founder, Jeff Bezos, “has sought to supplant humans with software since he was a mere bookseller. In one famous episode editors working on book reviews and recommendations were replaced by code that did the same work by mining shopping patterns” (Day, 2021). It also helps to note that, in their warehouses, Amazon currently supplements their use of robots and algorithms with a “plug-and-play [human] workforce that can be adjusted almost instantly”, and that Amazon’s long-term goal is to build fully automated systems that would enable them to better satisfy customer demands (Ibid). As suggested in the preceding paragraph, these developments aim to reduce both (1) Amazon’s labour (and total production) costs, and (2) the costs of its customers—who can spend less time working or shopping. Moreover, when noted alongside the two-century-long trend towards diminished work hours in high-income countries (Greenwood et al., 2021), such developments suggest that, in regard to productive capacities in general, humans may not be as essential as many believe.

The widespread presumption that humans are essential to certain activities is, by and large, underpinned by a belief in human exceptionalism: i.e. the idea that important human capacities are the result of non-routine processes that will never be fully explicable, and thus, never fully replicable by (human-designed) machines (e.g. Autor, 2015). As this belief rules out the possibility that the likes of Amazon could ever (3) help bring about a post-work world, it is important to note that there are at least two reasons for thinking that human exceptionalism is mistaken. First, as machines have proven capable of making routines out of more and more human activities (e.g. self-driving autos, translation technologies), it may well prove that we are more explicable, and more computationally replicable, than human exceptionalists presume (Susskind, 2018: 9). Second, it is not clear that machines need to replicate humans when completing tasks that people anthropocentrically consider humans as being uniquely capable of. Rather, machines could do things, and arguably already do a great many things, in their own machine-like ways that exceed our own human-like ways in terms of quality and/or quantity (e.g. Danaher, 2019: 43–47).

As Keynes (1930), Gorz (1985) and a long list of less-famous others have suggested, then, the emergence of a fully post-work world at some point in the future is a real possibility. But the fact that no one can confirm with 100% certainty that such a world will emerge, let alone provide exact dates as to when, should not obscure the recognition that technology has long enabled fewer people to do more with less, in different, and generally more intensified, ways. Moreover, the fact that such changes and potentialities are commonly highlighted by Big Tech luminaries who have clear motive to speed up the very changes and potentialities they are commenting on does not mean that such phenomena immediately become less real or important either.

The triumphalist tone, for example, that the incredibly rich former Chief Executive Officer (CEO) of Google, Eric Schmidt, adopted, when he suggested that “the coronavirus pandemic should teach Americans to be ‘a little bit grateful’ for powerful tech companies” (Schleifer, 2020), was clearly too much for many. Nevertheless, and galling as it may be, it is difficult to deny that at least some people’s lives have been made easier not just because of Amazon—which was singled out for praise by Schmidt (Ibid)—but because of Alphabet, Apple and Microsoft, too: whose computing hardware, online platforms and software, provide a key part of the remote (home) work infrastructure for many.

Despite remote work raising the spectre of never being able to leave “the office”, the rapid and widescale implementation of remote work during the COVID-19 pandemic—when it jumped from 5% to approximately 50% of full paid workdays in the United States (US)—appears to have been a broadly positive experience, with “average workers” hoping to keep working from home 46.5% of the time post-pandemic (Barrero et al., 2021). Potential explanations for this preference relate to workers being able to devote part of the time they save from less commuting to leisure and household activities, and to people appreciating the flexibility that remote work entails. And while the productivity of remote working is much contested—even by Big Tech companies who have historically spent huge amounts developing “campuses” that are presumed to provide “water cooler” fuelled innovation—current signs suggest that many tech companies will increasingly adopt, at a minimum, hybrid home/office work policies: for, like workers across the board, many who work for Big Tech do not want to go back to the office, or the “campus” in their case, full time (Axion, 2021).

Through their investments in technological advances that can displace human labour, and through their helping build the digital infrastructure that enables (at least some) people to work (and complete other chores) from home, Big Tech may be contributing to the proliferation of a less-work ethic (if not a full-blown anti-work one). In the first instance, this sort of less-work ethic relates to there being little to be gained (outside of cultural capital within hipster communities) from humans working to producing something “by hand” when it can be produced better and more cheaply by a machine. Second, it relates to remote work potentially resulting in people feeling at an increasing (emotional) distance from their co-workers, bosses and organizational employers. As many derive identity and social benefits from their work, this could prove a major source of discomfort, and could result in some experiencing what amounts to a complete “loss of self”. But it could ultimately prove of huge benefit as well, for it would likely open space within which people can form new identities and social relations that are separate from their externally motivated, and thus, alienating, work activities.

In such material and ideational ways, the world’s biggest and most powerful corporations could be helping to build “a capitalist road to communism” (van der Veen & van Parijs, 1986) where work and basic needs satisfaction are de-coupled. Nevertheless, in the short to medium term, any possibility of experiencing a less-work, let alone a post-work, world, are likely to be limited to populations or countries that are already relatively well-off: e.g. people in Finland, Japan, Spain, New Zealand, the United States, etc. Moreover, it is undeniable that at least some humans will be needed to perform vital tasks for the foreseeable future. As a result, any transition from a work world to a post-work world seems likely to exacerbate existing concerns regarding such matters as distributional justice and technocratic elites, and will likely be characterized by a stark divide between those workers who are “essential” to the provision of social goods and services, and those who are not, and all that such a divide would entail.

### Conclusion

In summary, the contributions that Big Tech companies are making to the potential emergence of a post-work world could significantly disrupt existing distributions of income, wealth, work, leisure time and so on. But over a longer time, these transformations would also seemingly result, if they are fully realized, in business and business ethics (which are very difficult to think of without the need to engage in [paid] work) being of little more than historical interest. While some are likely to celebrate such a possibility, others will find it a major cause for concern. Whatever the case, the perspective of Big Business Ethics helps bring such possibilities, and the role that the world’s largest corporations play therein, to the fore. If for no other reason, then, Big Business Ethics seems worthy of consideration because it points to developments that could result in business (ethics) as we currently know it, coming to an end.

## Ethical Concerns in Labour Relations Amidst Escalating Megatrends

**Ernesto Noronha and Premilla D’Cruz**

### Introduction

The International Labour Organization (ILO), in its recent report on the future of work, outlined four major megatrends: globalization; “greening” of the economy; changes in demographics; and technological advances, including artificial intelligence (AI), automation and robotics (ILO, 2019). These major ongoing transformations have implications for ethical issues that confront labour. This editorial commentary speaks to ethical issues that mark the labour-business interface vis-à-vis global production networks (GPNs), the environment, demography and, finally, technology. The underlying theme across these megatrends seems to be that, while labour is confronted with the issue of precarity in one form or the other, we provide a more nuanced understanding of the implications for business ethics.

### Global Production Networks

As is well known, the emergence of global production networks (GPNs) has been made possible by rapid advances in transport, data communications and information technology (IT), fragmenting production and enabling its relocation across international borders. Further, it was assumed that, if suppliers can increase profits through economic upgrading, social upgrading outcomes in terms of measurable standards (wages, benefits, etc.) and enabling rights (freedom of association, collective bargaining, etc.) would follow (Barrientos et al., 2011). While it is well established that GPNs have brought employment and economic growth to many developing economies, particularly in Asia, they are also associated with exploitative employment relations, environmental irresponsibility and recurrent ethical dilemmas (Clarke & Boersma, 2017). As shareholders, institutional investors and consumers comprehend and critique the social and ethical performance of corporate entities, businesses, to achieve more socially responsible decision-making and mitigate losses emanating from their social reputation (Wright, 2016), have subscribed to codes of conduct, multi-stakeholder initiatives (MSIs) and international framework agreements (IFAs) that improve working conditions and provide a living wage, promote freedom of association and collective bargaining, entail equality and non-discrimination, particularly with regard to gender, monitor health, safety and child labour, and abolish forced labour (Giaconi et al., 2021).

Notwithstanding efforts towards responsible business policies and practices, evidence suggests that ethical issues persist and these efforts have not generated sustainable improvements in the working conditions of workers (Kuruvilla et al., 2020), and this is particularly true for tier 2 and 3 firms (Islam & Stinger, 2018) and even for tier 1 suppliers operating in lead firm countries in the West (Noronha et al., 2020). The deliberate blurring of accountability to meet labour standards while proposing to remedy the same seems to be a major challenge. Further, these ethical dilemmas may have been exacerbated during the ongoing COVID-19 pandemic, particularly in countries of the Global South where informal labour had to endure harsh lockdowns without any support from employers or the state. In some cases, employers have tried to address these ethical challenges by designing programmes like the Social Compact. However, these experiments should not merely help businesses to inexpensively “bluewash” their reputation (see Noronha, 2022). Therefore, further research is required on how diverse sets of regulations coalesce together and are shared by all private and institutional actors (Dahan et al., 2021) to promote ethical labour practices. More importantly, a search for ways to be ethical should include the independent voice of labour in the workplace, thus far neglected, to make the different modes of regulations effective.

### The Environment

The ethical obligations of GPNs are further complicated by environmental governance issues (Singer & van der Ven, 2019). While the environmental case for switching to non-fossil energy seems irrefutable (Elliott, 2015), this will lead to a loss of jobs as economic activity and value added in high-emitting sectors is reduced (van der Ree, 2019). For instance, Neimark et al., (2020) argue that, as certain subsistence-level economic activities might become outlawed through increasingly stringent carbon control, a new eco-precariat may emerge. To illustrate, municipal authorities in New Delhi, India, have embraced waste-to-energy incinerators, while wastepickers fear that these changes threaten their access to waste (Demaria & Schindler, 2015), despite these workers already being poorly compensated, regularly stigmatized and frequently invisible in policy decisions (Gidwani, 2015). In fact, the ethics question becomes increasingly complicated as proponents of the green economy justify low or no pay, flexible working patterns and uninspiring work, while framing environmental work as morally rewarding (Castellini, 2019).

Notably, the 2015 United Nations Climate Change Conference negotiated some of the business ethics dilemmas posed by environmental issues by recognizing the importance of placing the interests of workers and communities at the forefront of decarbonization efforts so that “decent work” and “quality jobs” can be pursued simultaneously with climate action (While & Eadson, 2021), a development often referred to as the “just transition” (Kenfack, 2019). It is widely believed that the just transition purports to smoothen the shift towards a more sustainable society that respects the human and labour rights of those who lose jobs due to the abandonment of fossil-fuel-based work (Elliott, 2015; Kenfack, 2019). For instance, in the 1990s, when Germany dramatically reduced the burning of coal to generate electricity, it used widespread programmes to retrain coal industry workers to find new jobs, sometimes in renewable energy (Miller et al., 2013). At the same time, organizations will be ethically challenged to ensure that the new jobs created are “good jobs” and not only have decent working conditions, pay a living wage and provide clear career progression opportunities (Healy & Barry, 2017), but also hold room for social dialogue with workers and economy-wide skills development and retraining, buttressed by social protection and safety nets (While & Eadson, 2021). Besides this, the just transition agenda should challenge organizations to implement practices that are not only ethical in terms of the quality of jobs but also inclusive since all minorities and women are likely to be adversely impacted.

### Demography

Changes in demographics are no less significant in affecting the labour relations-business ethics link. On the one hand, the youth population is increasing in some parts of the world, while, on the other hand, the ageing population is increasing in other parts of the world. These developments put pressure on labour markets and social security systems. The ethical challenge is to balance the interests of youth populations with those of ageing populations. Regarding the latter, declining returns on pension investments and a reduced revenue base raise concerns about the sustainability of social protection systems (ILO, 2019). Workplace policies that were designed when life expectancy was lower than average retirement ages are no longer relevant in today’s workplace (Berger, 2021). As restrictions have been placed on the ability of employers to use a mandatory retirement age (Lain, 2017), providing support for a lifelong-active society would be an ethical way to alleviate the pressure on social protection systems (ILO, 2019). The issue of how to retain older workers in productive employment will be one of the most significant policy issues facing governments and organizations; this means devising humane policy alignments that assist older workers displaced by industry restructuring, enabling late-career transitions and providing flexible working arrangements (Gahan et al., 2017). Further, organizations will be ethically challenged to work around negative views about age and capacity in the workplace that have been unaffected by anti-discrimination laws or the right to request for flexibility (MacDermott, 2016). Ageism can have consequences such as traumatic or disturbing effects on the self-esteem and physical health of older workers, exposing them to an increased risk of depression (Berger, 2021). Responsible and sustainable policies on older workers should be designed with equal attention to their consequences for young workers (Gahan et al., 2017), who also deserve commensurate ethical treatment.

Young workers across the world experience several challenges, which entail business ethics implications, as they interface with the world of work. Despite high levels of education and technological skills, millennials across the developed world are plagued by high levels of unemployment and underemployment and have had to accept less-than-ideal employment, lower pay and fewer benefits (Ng et al., 2017). In the case of countries like India, the situation gets exacerbated as employers complain about the shortage of workers with requisite skills. The country is thus in a paradoxical situation where the number of educated unemployed seems to keep increasing, while employers across various sectors lament the lack of skilled human resources (Noronha & D’Cruz, 2021). Young people will also need help in transitioning from education to work (ILO, 2019). The ethical way out of this demographic challenge is to balance the needs of older workers with the aspirations of young workers without discriminating against either.

### Technology

Technological advances, particularly automation and digitalization undergirded by increasingly sophisticated artificial intelligence (AI) systems, hold enormous implications for work, employment and labour (Rogovsky & Cooke, 2021) and raise numerous ethical issues.

Modern ubiquitous IT technologies have been increasingly relied upon by workplaces since the last decade and, more recently, have facilitated the continuity of business through work-from-home during the COVID-19 pandemic (Mukherjee & Narang, 2022). These technologies entail new forms of control that are increasingly mobile, flexible, atomized, distant and informal, disguised within a rhetoric of emancipation and autonomy, which renders them subtle, insidious and misleading. Reflecting Lyon and Bauman’s “liquid surveillance” (Leclercq-Vandelannoitte, 2017: 143), which extends beyond organizations’ spatio-temporal boundaries and promises unprecedented organizational success, involves employees’ nearly continuous availability and responsiveness, information and cognitive overload as well as digital traceability, all of which imply heightened controls, cultures of permanent urgency and speed, a breakdown of personal and professional boundaries, reduced opportunities for respite and recovery and ill-health. The resultant loss of autonomy, invasion of privacy as well as stress and anxiety, which reduce human dignity within a context marked by systemic dilution of accountability and morality (Leclercq-Vandelannoitte, 2017), hold inevitable implications for business ethics. Questions which emerge include: What does the use of modern ubiquitous IT technologies imply for socially responsible and sustainable employee relations practices? What are the ethical implications for worker autonomy and privacy, meaning of work and physical and mental health, including time for detachment, repair and recovery?

Further, the platform economy, operating as pseudo-sharing, is known to exploit workers due to undesirable social conditions, including precarious jobs, insufficient incomes, powerful and unregulated corporations, and worsening inequality, accompanied by stringent technological controls (Chai & Scully, 2019), discrimination and abuse (D’Cruz & Noronha, 2018). Ethical concerns clearly define platforms. Not only are workers termed “independent contractors”, absolving platforms of employer responsibilities, but platforms largely operate beyond the purview of regulation and democratic oversight (D’Cruz & Noronha, forthcoming). While workers interact with a technological application such that there is no appearance of power, the invisibility and obscuring of capital and its modus operandi and gains, the global spatial dispersion and competitive entrepreneurial individualism of and transfer of costs and risks to labour, and the limits to workers’ collective negotiation strength and contestation opportunities evidence how precarity undergirds exchange (Chai & Scully, 2019; D’Cruz & Noronha, forthcoming). The fundamental ethical question which arises is how can platforms develop into accountable and responsible entities which provide “good jobs” and guarantee “decent work”, such that worker rights are sustainably safeguarded? Relatedly, how can socially responsible regulatory mechanisms commensurate with platforms’ geographical reach be designed and executed? Further, how can platform capitalism’s ethical practices encompass sustainable employee relations practices, including worker collectivization and negotiation along known and re-imagined lines?

The rise of AI and robots have sparked new ethical challenges for business and society (Morse et al., 2021). Shrinking labour markets due to increasing reliance on AI and robotization, which replace humans, displace the latter from work, reducing job opportunities (D’Cruz & Noronha, 2021). Though some highly skilled workers are slated to succeed in this new environment, far more are expected to be displaced into lower-paying jobs at best or permanent unemployment at worst, fuelling job insecurity, precarity and ill-being (D’Cruz & Noronha, 2021). Kim & Wolf (2019) invoke Keynes’s “technological unemployment” to describe the situation. Yet how does technological unemployment interface with ethical business practices and what is the role of governments, business and civil society here? Importantly, technological unemployment, even if accompanied by guaranteed basic income, raises ethical issues because it precipitates axiological challenges which, in turn, could lead to teleological challenges. The former implies that technological unemployment will leave many humans without the opportunity to add meaning to their lives through work, depriving them of a sense of fulfilment linked to contributing to the larger social good, and thereby resulting in a crisis where human dignity is at stake. The latter implies that, while automation maximizes firm efficiency, effectiveness and profits, the question remains as to whether it is ethically desirable for corporate purpose and governance to endorse such an approach, given the axiological challenges involved (Kim & Wolf, 2019). What does state guarantee of basic income in instances of technological unemployment imply for sustainable business, responsible management and worker rights? Crucially, the overarching question arising is how are ethical employment relations practices impacted by technological innovation and its implications for employment?

As workplaces rely increasingly on robotization, “workplace trans-entity bullying” where robots mistreat humans is reported (D’Cruz & Noronha, 2021: 295). AI and robots may be programmed for accuracy, with fairness and inclusion being overlooked (Morse et al., 2021). Indeed, AI and robots are known to display preferences for particular social identities (e.g. race, gender and age), giving rise to discrimination which can evolve into category-based harassment and bullying (D’Cruz & Noronha, 2021). Then again, whereas AI and robots facilitate efficiency, making task execution quicker (Wilson & Daugherty, 2018), this leads to work intensification (Gostautaite et al., 2019), such that when the pace is stepped up, humans can feel the effects of stronger controls and even exploitation. Indeed, some humans speak of “running” all the time to keep up with their robot co-workers (Gostautaite et al., 2019). If humanoids controlling humans at work (Frick, 2015) go on to behave abusively in the pursuit of the organizational agenda, depersonalized bullying could well result (D’Cruz & Noronha, 2021). Moreover, social-assistive robots deployed in healthcare settings can bully their human care recipients through exclusion, name-calling, insults, threats and manipulation (Coffee-Johnson & Perouli, 2019). The abuse of human care recipients by robot caregivers is unfortunate, given that social-assistive robots, increasingly relied on for caregiving assistance, have been seen as alternatives to human caregivers in the healthcare system, based on reports that the latter abuse their human care recipients (Frennert & Ostlund, 2014). As technological progress in AI and robots unfolds, how can ethical considerations define the business agenda? That is, AI and robots designed for workplaces should be programmed for sustainable employment relations with human workers. In other words, how can relational dignity and social responsibility mark AI and robots at work such that exploitation, discrimination, emotional and physical violence, destruction and other wrong-doing towards human workers are eliminated? As advanced general intelligence evolves and robots increasingly acquire growing physical and social agency, autonomy and skills, (a) how can they be armed with a sense of discernment that facilitates their morally appropriate and socially responsible behaviour at work, and (b) what sense of legal and moral entitlement should they be accorded at work?

### Conclusion

The transformative potential of the four megatrends highlighted in this editorial commentary should be harnessed to create decent and sustainable work for all. It should address issues such as how workplaces can reconcile their quest for competitive advantage alongside safeguarding worker rights; what workplaces must do to enact responsible goals, policies and practices which endorse and perpetuate sustainable employment relations focused on protecting human capital; and how workplaces can ensure that their goals, policies and practices reflect and strengthen the UN SDGs (United Nations Sustainable Development Goals), with a particular view to safeguarding worker interests. In this regard, the ILO’s (2019) human-centred agenda seeks to steer the ongoing transformations towards work that affords dignity, security and equal opportunity, expanding human freedoms in the future. “It means guaranteeing fundamental rights at work, ensuring that all workers are afforded adequate labour protection, and actively managing technology to ensure decent work.” (ILO, 2019: 28).

## Religion, Spirituality and the Workplace: Anticipating the Next 40 Years

**K. Praveen Parboteeah**

### Introduction

Although various scholars have argued that religions' influences on societies will decline because of the weakening effects of modernization and secularization on religion, current scholarship shows that religion remains influential in the workplace (Van Buren et al., 2020). Religion, the set of shared beliefs and institutions based on faith in supernatural forces (Parboteeah et al., 2009), will likely continue to play an important societal role. Furthermore, despite some differences, scholarship in the related concept of spirituality (Chan-Serafin et al., 2013) also stays strong.

As the Journal of Business Ethics celebrates its 40th year of existence, it becomes critical to assess some of the significant workplace changes and how religion will impact or mitigate them over the next 40 years. Experts project growth in informal and precarious jobs, and an ageing working population in high-income countries and a much younger population in middle- to low-income countries (Abeliansky et al., 2020), rising inequality because of wage gaps and concentration of corporate power (Grimshaw, 2020), and increased use of technology and artificial intelligence (AI) (Jain et al., 2021). These changes should all make workers feel more disengaged from their workplace. However, the rising inequality and increased use of AI likely make workers less connected with each other, thereby resulting in anomie or a sense of loneliness.

Religion can play a positive role in helping employees face these challenges, as religious “practices that unite people irrespective of colour, race or nationality may lead to organizational practices that draw out the positive nature laden in individual employees and managers” (Van Buren et al., 2020: 805). An examination of the trends in religion also suggests the growth of Islam and Hinduism over the next few decades (Pew Research Center, 2015), thereby suggesting future research questions in those affiliations. However, the future also implies that religions may also play negative roles, as seen through their role in furthering discrimination in the workplace (Prasad et al., 2020), and other adverse psychological effects (Chan-Serafin et al., 2013).

This commentary will offer a balanced view of religion, emphasizing religion’s future role related to business ethics. Such roles will be examined in the light of current research to provide research suggestions for the next 40 years. The commentary is structured as follows. I first discuss these trends and present preliminary observations of how these changes should impact scholarly research on religion. I argue that religion will continue to remain strong in the workplace, but will continue to positively and negatively affect the workplace. In the light of such trends, I argue that religion scholars will also need to start acknowledging the conflicting impact of religions in the workplace. I discuss some of the critical themes these aspects represent. I also discuss the implications of such themes and avenues for future research.

### Major Trends and Implications

Abeliansky et al. (2020) argue that workplaces worldwide will see important changes, including a significant increase in younger workers entering middle- and low-income countries and more workers involved in informal jobs and the underground economy. Experts also suggest that workers will continue to be hounded by rising inequality because of wage gaps and the concentration of corporate power (Grimshaw, 2020). Such trends have important implications for scholarship on religion in the workplace. First, there is no doubt that religions will continue to influence the workplace in the next 40 years. Workplace changes such as the sustained growth of informal jobs and income inequality will mean that workplaces will continue to experience poor working conditions that make workers feel even more disengaged from their jobs. Furthermore, it is clear that automation may replace many of the lower-skilled jobs that lower-income countries' workforce depends on. Given that such countries provide many manufactured products such as clothing that higher-income countries rely on, and that workforce growth will occur in these countries, it is very likely that workforces will face even more dehumanized workplaces. Religion will continue to play important roles, because it is recognized that religions can benefit workers. As we discuss later, religions can provide solace in the workplace.

A second significant trend is the growth in artificial intelligence (AI) in the workplace (Jain et al., 2021). Although AI can make life better by integrating human and computer intelligence to solve societal problems, the focus on performance rather than intelligence suggests important roles that religions can play. Consider, for instance, the use of AI to select job candidates, predict fraud, approve loans and protect privacy. In such cases, the design of such systems depends on the algorithmic principles based on the quality of the data used and human authority. As such, religions can provide important principles as companies design new AI systems. Consider, too, that the development of AI systems and other automated system design is dependent on human authority, or the entities that provide the communication to develop such systems (Cheong, 2021). However, while some experts have advocated a more techno-centric approach to AI design devoid of human values, given the importance of religion at the workplace it may be fruitful to companies to incorporate religious views and values as AI systems are being developed. For example, consider the development of the robot monk at the Longquan Monastery in Beijing (Cheong, 2021). The monastery worked with a technology company to develop a robot that “can explain Buddhist tenets, chant mantras, sense its environment…” (Cheong, 2021: 13). The process involved religious officials working closely with the system’s designers to ensure that the human element and values were present in the robot monk. Development of the robot involved close cooperation where religious leaders saw science as inevitable rather than a schism with religion. Similarly, as companies develop algorithms for decision making, they can consult with religious leaders to ensure that they know the inherent design principles and whether such principles violate ethical codes or other human rights. Religion can thus provide the guiding principles to make AI systems that incorporate human values.

A third important trend is that higher-income countries will see a growth of older workers, while it is also recognized that companies may have difficulties in retaining such workers (Abeliansky et al., 2020). Hence, given the potential of religion to provide some solace to workers, religion will continue to offer buffering effects to help older workers in these countries to cope with work demands. Given the high percentage of religious individuals at work in high-income countries, research can examine whether those companies that allow stronger integration of religion at the workplace can attract and retain more skilled older workers. Additionally, recent developments in the Faith and Work Integration Scale (Miller et al., 2019) provide interesting research avenues, because researchers can examine the impact of the integration of religion on business ethics issues.

However, this trend may pose a potential for conflict arising, since middle- to high-level income countries will also likely see a growth of employees who do not affiliate with any religion (Pew Research Center, 2015). This growth suggests that companies may have to contend with managing the conflicting effects of religion. Such changes indicate that higher-income countries may see conflicting influences of religions. On the one hand, companies may need to work harder to retain older workers, and integrating religion in the workplace may become a factor. At the same time, those employees who do not identify with any religion may resent the more robust integration of religion in the workplace. Such trends also suggest that more companies may have to balance integration with increasing the number of agnostic or unaffiliated workers. Future research will also need to understand how Faith and Work Integration may also result in some dissatisfied or less engaged workers. Furthermore, as we discuss later, the interaction of minority and majority religions within companies also suggests interesting research avenues.

In addition to understanding the implications of the above workplace changes, it is important to know how the religious landscape is changing. The most recent report by the Pew Research Center (2015) suggests several important trends. Although Christianity will remain the largest religious group globally, Islam will see the highest growth of all religions worldwide. Current trends suggest that the number of Muslims will likely be equal to the number of Christians in 2050. Furthermore, except for Buddhism, all other religions such as Hinduism, Judaism and folk religions, will grow significantly. However, most of Europe and North America will see a growth of individuals who identify as atheists, agnostics or do not affiliate with any religions.

The growth of specific religions such as Islam and Hinduism also has important implications for future religious scholarship. Given that religion research has overwhelmingly taken a Christian perspective, such growth suggests that domestic companies and multinationals will contend increasingly with employees who follow the Islamic or Hindu faiths. Religion scholars will need to provide more insights into such faiths. As we discuss below, such trends have important implications for the workplace and business ethics.

### Research Directions for the Next 40 Years

Given the above trends, what shape should religion scholarship take over the next 40 years? It is firstly important to understand the progress that has been made. Although there was once hesitation in conducting research on religion in the social sciences, the last two decades have seen significant progress. The Journal of Business Ethics has published numerous articles on religion: a review of articles since the creation of the Journal shows that more than 100 articles have been published examining the interface of religion and the workplace. The Journal has also published more than 50 articles in related spirituality research. A review of articles also shows that the Journal has published articles on specific religions such as Buddhism, Christianity and Islam. Surprisingly, there are fewer articles on Hinduism. Additionally, recent research has provided theoretical frameworks and empirical conceptualizations to understand religion and ethics (Parboteeah et al., 2008) and how religions relate to the workplace (Parboteeah et al., 2009), thereby also proposing operationalization of religions that go beyond belief in God. Religion’s important influences on business ethics issues has also been recognized by Van Buren et al. (2020) in a special issue in Business & Society.

In the light of the above progress, several important future research themes emerge. First, the workplace will see increasing diversity. Domestic and international companies will see more individuals of different religions interacting. Such changes suggest that future scholarship will need to start examining the effects of religion on aspects of diversity in the workplace. While this seems a cliché, given the push towards more understanding of diversity in the workplace, it is surprising to see that extant studies have mostly ignored this link. However, the push towards diversity and inclusion is relatively new and will likely continue. Future scholarship is encouraged to examine how religions can contribute to diversity efforts in companies. Most religions advocate respect for others, and such an assumption suggests that religion should potentially have positive effects on the diversity climate in the organization. Consider Braunstein et al. (2014) ethnographic study and their findings that the practice of daily prayers helped bridge differences in groups within faith-based community organizations. Such research showed that joint prayer practices associated with religion helped manage the organizational challenges associated with racial and socio-economic diversity. Religion can play a positive role in assisting employees in face these challenges, because religious “practices that unite people irrespective of colour, race or nationality may lead to organizational practices that draw out the positive nature laden in individual employees and managers” (Van Buren et al., 2020: 805). It is hoped that future scholarship will identify the aspects of religion such as prayers, meditation and reflection that can potentially enhance diversity management within the organization.

Despite the above, it is undeniable that religions can also be very divisive and hurt diversity efforts. Consider the current rise of Indian nationalism and the consequent impact on Muslim minorities. Researchers are therefore encouraged also to understand the divisive potential of religion. One possibility in this area is the consideration of religious plurality or the degree to which people from different religious affiliations must co-exist in a social system. Religious plurality has received attention at the country level (Parboteeah et al., 2009) but has been mostly ignored within organizational contexts. Future scholarship should be open to examining religious plurality within organizations and the potential of such plurality to negatively impact organizational outcomes. However, if managed well, it is also possible that religious plurality can have potential advantages such as more creative and innovative companies. The diversity of religious beliefs inherent in religious plurality also suggests the potential for religion to provide diversity of thoughts. It is hoped that this commentary will encourage scholarship in that direction.

In addition to the above, the increased diversity of religious affiliations should spur other interesting avenues within the international business realm. Consider the possibility that multinationals with Christian headquarters may increasingly contend with employees in emerging markets where the latter are affiliated with other religions. Furthermore, it is possible for emerging market multinationals’ supervisors to have to manage employees with other religious affiliations. Diversity research will need to understand the implications of such interactions. For example, the future may see more expatriates causing friction as they manage employees of different religious backgrounds. The expatriate literature has also seen a dearth of studies related to religion, and future scholarship needs to understand how such international assignments may create friction. Furthermore, extant research suggests that the faith of expatriates has been mostly ignored. It is hoped that research questions related to religious expatriates and the many other research questions related to the international business literature as it manages diversity, can be examined.

Another potential research area related to the above trends is the potential interaction of majority and minority religions within multinationals and the potential impact on employees. It is feasible to see how majority religions may create conflicts with the minority affiliated employees. As such, it is hoped that future scholarship will also investigate such potential for conflict. According to Syed & Ali (2021), minority religions often face a “pyramid” of hate based on bias, discrimination and violence. Such issues are deserving of scholarly attention, as researchers need to understand the impact of majority religions on minority workers. In terms of methodological approaches, it is essential to acknowledge that religion research has favoured more quantitative research approaches. However, the potential interaction of employees from minority and majority religions and the evolution of such interactions lend themselves to qualitative approaches. Therefore, it is hoped that this commentary will inspire more research using such qualitative approaches. Consider, for instance, Pandey & Varkkey’s (2020) interviews with trade union members to see how caste membership impacts trade union workers in state-owned companies in India. Qualitative approaches such as those will likely provide important insights into the complexity and subtleties of the religious phenomenon under study.

Although the interaction of majority and minority religions may result in conflict, it is also important for future researchers to address the potential for positive outcomes because of such interactions. Minority religions can sometimes be a source of positive changes. Consider Vaidyanathan’s (2020) examination of Roman Catholicism in Bangalore and Dubai. The study shows how macro-level manifestations of the minority religion impact its members and wider society. As mentioned earlier, the minority religion allowed its adherents to better cope with work demands while also providing important social capital for adherents to flourish. However, most importantly, the qualitative study also showed how the minority religion had an impact on capitalism in both cities through its impact on how its adherents approached many issues, including those with moral implications. In this case, the minority religion positively impacted the social institutional environment. There is no doubt that there is a possibility that the majority religion can also positively influence the minority religion, and it is hoped that future scholarship will consider such potential.

Second, an important trend that is now affecting most companies is the use of AI and other automated systems. As argued by Jain et al. (2021: 677), repetitive tasks such as “in warehouse, assembly lines, and fast-food restaurants, have been early targets for automation because it is relatively easier to capture quality data in such task scenarios”. Such systems do not necessarily pose moral implications. However, identifying fraudulent transactions, selecting job applicants, etc., and other aspects pose more challenges, since they depend on algorithmic processes that can introduce biases and other elements of injustice. While there has been some philosophical discussion of the relationship between religion and AI (Singler, 2020), future scholarship needs to start considering more practical empirical considerations of the role of religion. Most religions provide essential prescriptions about morality, and such aspects need to be considered by companies as they continue relying on AI. As AI moves into the realms of what are deemed high-stake applications, such as self-driving cars, and are used in military applications, the development of algorithms would benefit from consideration of religious principles. As such, religions can play critical roles in this realm. AI faces many challenges, such as which aspects of decision making to automate and how to avoid biases in the algorithms. Religion can become the source of moral guidance to tackle these challenges. Similarly, the related discipline of spirituality can also become a source of moral guidance. At the same time, religions can also be used to justify algorithms that discriminate against other minorities. Researchers must also tackle such possibilities.

Third, it is also possible that many other recent changes, such as working from home, use of virtual meeting platforms such as Zoom, and automation, may also encourage more worker disengagement. Such processes may likely result in anomie within the workplace, where workers feel disconnected from each other and do not find meaning in their work. Religions can be useful to counteract such negative influences. Consider the earlier-mentioned research by Vaidyanathan (2020: 898), who found that members of the Roman Catholic Church looked forward to weekly prayers since “religion can enable corporate life by serving as a refuge providing worn-out professionals a means of rejuvenation and release”. Furthermore, the related spirituality research stream may also provide the potential for future research questions. As argued by recent research, workplace spirituality “nourishes employees’ spiritual needs and provides them with an opportunity to grow” (Lata & Chaudhary, 2021). Therefore, it can provide good buffering forces to help employees deal with such changes. Future research should investigate such possibilities.

Fourth, future research should start focusing more on understanding the religions of Islam and Hinduism. A review of research published in the Journal of Business Ethics shows about 40 articles examining the link between Islam and key outcomes such as corporate social responsibility, job outcomes and other key organizational outcomes. Not surprisingly, such research has focused on financial aspects, given the prohibition of payment of interest in Islam. Additionally, most research has focused on neutral or positive aspects of Islam. Such scholarship should continue to be encouraged, given the many positive prescriptions emanating from Islam. Consider Gumusay’s (2019) discussion of the role of religion with a focus on Islam and other Abrahamic religions in leadership theories. Islam has many important facets that can be integrated to enhance ethics in an organization. For example, the concept of *akhlaq* presents significant potential for understanding how tenets of Islam can affect the workplace and ethics.

Although Islam has received significant scholarship, in contrast, a review of Hinduism research published in the Journal of Business Ethics reveals a significant void. As the world sees an increasing Hindu population worldwide, it will become more important to understand Hinduism and its implications for employees within a Hindu environment. While the field is aware of many Islamic principles, such as the prohibition of payment of interest and even the existence of the Islamic work ethic, Indian scholars have been more likely to devote scholarship to understanding spirituality. However, Hinduism has many interesting aspects that are deserving of inquiry. Hindus believe in the four stages of life, ranging from being a student to being a householder on to being in a liberated phase. Multinationals are well advised to appreciate such stages in the lives of their employees, as these stages have important implications for business ethics. Furthermore, with the growth of Indian populism, it is expected that Indian workplaces may see more integration of Hinduism in the workplace. Research to understand the impact of Hinduism on the workplace and the potential marginalization of Muslim workers and other minorities is sorely needed.

As the popularity of both Islam and Hinduism grows, it is also important for future scholars to adopt a balanced research agenda. Most research on Islam has tended to adopt a more neutral position on such research. However, it is recognized that Islam accepts that “a man is responsible for economically supporting his family members, including his wife and children while placing a high value on a woman’s role as mother” (Syed & Van Buren, 2014: 252). As Western-based multinationals continue to operate in societies with a high Islamic population, such teachings’ impact needs to be assessed. For example, we do not know how such multinationals balance the need to be culturally sensitive while also respecting headquarters’ norms for gender equality. Scholarship is sorely needed to understand these more detrimental aspects, while also understanding how multinationals can better manage these duelling pressures. However, it should also be noted that there are many interpretations of the Islamic faith, and not all view the role of women as subordinate to men. Consider Tlaiss’s (2015: 859) research, which showed how women entrepreneurs used their Islamic faith to “construct and navigate their entrepreneurial careers away from the traditional, doctrinaire interpretations of Islam”. Future research should be cognizant of such subtleties.

Similarly, Hinduism includes some principles that are often seen as lacking fairness and justice. Consider some interpretations of Hinduism that view women’s position in society as playing a more subordinate role in society. Recent research also acknowledges the role of Hinduism in perpetuating casteism in Indian organizations (Pandey & Varkkey, 2020) and is also seen in furthering discrimination in the workplace (Prasad et al., 2020). Noronha (2021) shows that such casteism is also prevalent in countries with a significant South Asian diaspora. Such research shows how even when members of the lowest caste (*Dalits*) move to other countries such as the US (United States) and the UK (United Kingdom), and achieve economic and political mobility, they are still stigmatized by *non-Dalits*. This shows that, despite globalization and other forces to integrate *Dalits* into the contemporary business environment, the effects of caste remain powerful and pervasive.

The next few decades should see more research examining the detrimental effects of Hinduism within companies. Furthermore, as the number of Hindus grows in US society and elsewhere, it is essential to assess how caste-based principles are being addressed. As Noronha (2021) states, more research is also needed in countries with a South Asian diaspora to understand the pervasiveness of casteism. Such research should be helpful to facilitate a more equitable view of individuals from different castes. As pressures continue for multinationals to treat all genders and occupations equally, such research can provide important insights. However, such research should also be aware of diverse interpretations of the Hindu faith.

A stronger focus on both Islam and Hinduism to cater to the growth of these religions also underscores the need for more multilevel research examining religion and organizational outcomes. Most research in the Journal of Business Ethics has been conducted at single levels of analysis. However, as the above discussions imply, an adequate investigation of religion’s effects requires consideration at different levels. Consider, for instance, the possibility of a Western-based or emerging-market multinational addressing equality through espoused principles. Such a scenario involves understanding the pressures coming from the international community (country level) on the corporate culture (firm level) and its impact on changing religious views (individual level). Such multilevel research will incorporate more complex models while providing further insights into these critical aspects.

### Conclusion

It is undeniable that religion will continue to play essential roles in the workplace. It is hoped that this commentary will inspire some future exciting research avenues. Additionally, while the commentary discussed the role of religion in general, the Journal of Business Ethics focuses on understanding business ethics issues. Many of the phenomena discussed pertain to ethical aspects of the organization. As co-editor of the Religion, Spirituality and Business Ethics section, I look forward to welcoming articles that address some of the big questions that will face us in the next 40 years. It is likely that all major religions of the world, such as Christianity, Judaism, Buddhism, Islam and Hinduism, have important commonalities that can be used to further business ethics and business ethics education (Ruhe & Lee, 2008). However, religions can also be detrimental to organizational life (Chan-Serafin et al., 2003). It is hoped that this commentary will also provide for a balanced view of religions at the workplace for the next four decades.

## References

[CR1] Abeliansky AL, Algur E, Bloom DE, Prettner K (2020). The future of work: Meeting the global challenges of demographic change and automation. International Labour Review.

[CR2] Andrews, D., Criscuolo, C., & Gal, P. N. (2016) The best versus the rest: The global productivity slowdown, divergence across firms and the role of public policy. OECD Productivity Working Paper No 5 OECD Publishing, Paris

[CR3] Autor D (2015). Why are there still so many jobs? The history and future of workplace automation. Journal of Economic Perspectives.

[CR4] Axion, S. 2021. Big tech companies are at war with employees over remote work. *Ars Technica*, 1 August. https://arstechnica.com/gadgets/2021/08/vaccines-reopenings-and-worker-revolts-big-techs-contentious-return-to-the-office/.

[CR5] Barrero, J. M., Bloom, N., & Davis, S. J. (2021). Why working from home will stick. NBER Working Paper 28731. Cambridge, MA: National Bureau of Economic Research

[CR6] Barrientos S, Mayer F, Pickles J, Posthuma A (2011). Decent work in global production networks: Framing the policy debate. International Labour Review.

[CR7] Berger E (2021). Ageism at work.

[CR8] Bostrom N (2014). Superintelligence: Paths, dangers, strategies.

[CR9] Braunstein R, Fulton BR, Wood RL (2014). The role of bridging cultural practices in racially and socioeconomically diverse civic organizations. American Sociological Review.

[CR502] Castellini V (2019). Environmentalism put to work: Ideologies of green recruitment in Toronto. Geoforum.

[CR10] Cave, S., Coughlan, K., & Dihal, K. (2019) Scary robots: Examining public responses to AI In *Proceedings of the 2019 AAAI/ACM Conference on AI, Ethics, and Society* (pp. 331–337).

[CR11] Chai S, Scully MA (2019). It’s about distributing rather than sharing: Using labor process theory to probe the “sharing” economy. Journal of Business Ethics.

[CR12] Chan-Serafin S, Brief AP, George JM (2003). Perspective—How does religion matter and why? Religion and the organizational sciences. Organization Science.

[CR13] Cheong PH (2021). Bounded religious automation at work: Communicating human authority in artificial intelligence networks. Journal of Communication Inquiry.

[CR14] Clarke T, Boersma M (2017). The governance of global value chains: Unresolved human rights, environmental and ethical dilemmas in the apple supply chain. Journal of Business Ethics.

[CR15] Coffee-Johnson, L., & Perouli, D. (2019). Detecting anomalous behavior of socially assistive robots in geriatric care facilities. *Proceedings of the 2019 ACM/IEEE International Conference on Human-Robot Interaction (HRI)* (pp. 582–583).

[CR16] D’Cruz P, Noronha E (2018). Abuse on online labour markets: Targets’ coping, power and control. Qualitative Research in Organizations and Management: An International Journal.

[CR17] D’Cruz P, Noronha E, D’Cruz P (2021). Workplace bullying in the context of robotization: Contemplating the future of the field. Concepts, approaches and methods, Handbooks of workplace bullying, emotional abuse and harassment.

[CR501] D’Cruz, P., & Noronha, E. (forthcoming). India’s platform economy experience: A site for the commodification-decommodification dynamic. In I. Ness (Ed.), *Platform labour and global logistics: A research companion*. Routledge.

[CR18] Dahan Y, Lerner H, Milman-Sivan F (2021). Shared responsibility and labor rights in global supply chains. Journal of Business Ethics.

[CR19] Danaher J (2019). Automation and utopia: Human flourishing in a world without Work.

[CR20] Day, M. (2021). In Amazon’s flagship fulfillment centre, the machines run the show. *Bloomberg Businessweek,* 21 September 21. https://www.bloomberg.com/news/features/2021-09-21/inside-amazon-amzn-flagship-fulfillment-center-where-machines-run-the-show.

[CR503] Demaria F, Schindler S (2015). Contesting urban metabolism: Struggles over waste‐to‐energy in Delhi, India. Antipode.

[CR21] Donaldson T (1982). Corporations and morality.

[CR22] Du S, Xie C (2021). Paradoxes of artificial intelligence in consumer markets: Ethical challenges and opportunities. Journal of Business Research.

[CR23] Elliott D, Hersh M (2015). Green jobs and the ethics of energy. Ethical Engineering for International Development and Environmental Sustainability.

[CR24] Flyverbom M, Deibert R, Matten D (2019). The governance of digital technology, big data, and the internet: New roles and responsibilities for business. Business & Society.

[CR25] Freeman RE (1984). Strategic management: A stakeholder approach.

[CR26] Frennert S, Östlund B (2014). Seven matters of concern of social robots and older people. International Journal of Social Robotics.

[CR27] Frick, W (2015) When your boss wears metal pants. *Harvard Business Review,* 84–89.

[CR28] Friedman B, Nissenbaum H (1996). Bias in computer systems. ACM Transactions on Information Systems (TOIS).

[CR29] Friedman M (1970). The corporate social responsibility is to increase its profits. New York times Magazine.

[CR30] Gahan P, Harbridge R, Healy J, Williams R (2017). The ageing workforce: Policy dilemmas and choices. Australian Journal of Public Administration.

[CR31] Giaconi M, Giasanti L, Varva S (2021). The value of “social” reputation: The protection of MNE workers from the consumer’s perspective. Global Jurist.

[CR32] Gidwani V (2015). The work of waste: Inside India's infra-economy. Transactions of the Institute of British Geographers.

[CR33] Gorz A (1985). Paths to paradise: On the liberation from work.

[CR34] Goštautaite, B., Luberte, I., Buciuniene, I., Stankeviciute, Z., Staniškiene, E., Trish, R., & Antonio, M. (2019) Robots at work: How human-robot interaction changes work design. Paper presented at the EAWOP Conference, June, Turin.

[CR35] Greenwood, J., Guner, N., & Marto, R. (2021) The great transition: Kuznets facts for family-economists. NBER Working Paper 28656. Cambridge, MA: National Bureau of Economic Research.

[CR36] Grimshaw D (2020). International organisations and the future of work: How new technologies and inequality shaped the narratives in 2019. Journal of Industrial Relations.

[CR37] Gumusay AA (2019). Embracing religions in moral theories of leadership. Academy of Management Perspectives.

[CR39] Healy N, Barry J (2017). Politicizing energy justice and energy system transitions: Fossil fuel divestment and a “just transition”. Energy Policy.

[CR41] Howard, A., & Isbell, C. (2020). Diversity in AI: The invisible men and women. *MIT Sloan Management Review 62*(2), 21 September. https://sloanreview.mit.edu/article/diversity-in-ai-the-invisible-men-and-women/.

[CR504] ILO (2019). Work for a brighter future: Global Commission on the Future of Work.

[CR42] Islam G (2021). Business ethics and quantification: Towards an ethics of numbers. Journal of Business Ethics.

[CR43] Islam MT, Stringer C (2018). Challenges of achieving social upgrading in Bangladesh’s apparel industry. Society and Business Review.

[CR44] Jain H, Padmanabhan B, Pavlou PA, Raghu TS (2021). Editorial for the special section on humans, algorithms, and augmented intelligence: The future of work, organizations, and society. Information Systems Research.

[CR45] Johnson, D. G. (1985). *Computer Ethics*. Englewood Cliffs NJ: Prentice

[CR46] Johnson DG, Floridi L (2004). Computer ethics. The Blackwell guide to the philosophy of computing and information.

[CR47] Kenfack CE (2019). Just transition at the intersection of labour and climate justice movements: Lessons from the Portuguese Climate Jobs campaign. Global Labour Journal.

[CR48] Keynes, J. M. (1930). Economic possibilities for our grandchildren. In J. M. Keynes (2010/1931), *Essays in Persuasion*, pp.321–332. Houndmills: Palgrave Macmillan

[CR49] Kim TW, Scheller-Wolf A (2019). Technological unemployment, meaning in life, purpose of business, and the future of stakeholders. Journal of Business Ethics.

[CR50] Kroll JA (2018). The fallacy of inscrutability. Philosophical Transactions of the Royal Society a: Mathematical, Physical and Engineering Sciences.

[CR51] Kuruvilla S, Liu M, Li C, Chen W (2020). Field opacity and practice-outcome decoupling: Private regulation of labor standards in global supply chains. ILR Review.

[CR52] Lain D, Parry E, McCarthy J (2017). Employment of workers aged 65 and over: The importance of policy context. The Palgrave handbook of age diversity and work.

[CR53] Lata M, Chaudhary R (2021). Workplace spirituality and experienced incivility at work: Modeling dark triad as a moderator. Journal of Business Ethics.

[CR54] Leclercq-Vandelannoitte A (2017). An ethical perspective on emerging forms of ubiquitous IT-based control. Journal of Business Ethics.

[CR55] Lutz C (2019). Digital inequalities in the age of artificial intelligence and big data. Human Behavior and Emerging Technologies.

[CR56] MacDermott T (2016). Older workers and requests for flexibility: A weak right in the face of entrenched age discrimination. Federal Law Review.

[CR57] Markets and Markets. (2021). Artificial intelligence market by offering (hardware, software, services), technology (machine learning, natural language processing), deployment mode, organization size, business function (law, security), vertical, and region - Global forecast to 2026. https://www.marketsandmarkets.com/Market-Reports/artificial-intelligence-market-74851580.html.

[CR58] Martin K (2019). Ethical implications and accountability of algorithms. Journal of Business Ethics.

[CR59] Martin K (2022). Algorithmic bias and corporate responsibility: How companies hide behind the false veil of the technological imperative. The Ethics of Data and Analytics.

[CR60] Mearsheimer JJ (2001). The tragedy of great power politics.

[CR61] Miller CA, Iles A, Jones CF (2013). The social dimensions of energy transitions. Science as Culture.

[CR62] Miller DW, Ewest T, Neubert MJ (2019). Development of the integration profile (TIP) faith and work integration scale. Journal of Business Ethics.

[CR63] Moore G, Spence L (2006). Editorial: Responsibility and small business. Journal of Business Ethics.

[CR64] Morse L, Teodorescu MHM, Awwad Y, Kane GC (2021). Do the ends justify the means? Variation in the distributive and procedural fairness of machine learning algorithms. Journal of Business Ethics.

[CR65] Mukherjee S, Narang D (2022). Digital economy and work-from-home: The rise of home offices amidst the COVID-19 outbreak in India. Journal of the Knowledge Economy.

[CR66] Neimark B, Mahanty S, Dressler W, Hicks C (2020). Not just participation: The rise of the eco-precariat in the green economy. Antipode.

[CR67] Ng ES, Lyons ST, Schweitzer L, Parry E, McCarthy J (2017). Millennials in Canada: Young workers in a challenging labour market. The Palgrave handbook of age diversity and work.

[CR68] Noronha E, D'Cruz P, Noronha E, Caponecchia C, Escartín J, Salin D, Tuckey MR (2021). Caste and workplace bullying: A persistent and pervasive phenomenon. Handbooks of Workplace Bullying, Emotional Abuse and Harassment, Vol 3 - Dignity and Inclusion at Work.

[CR505] Noronha E (2022). Social Compact: Co-creating socially responsible businesses the Indian way. Vikalpa.

[CR69] Noronha E, D’Cruz P, Rogovsky N, Cooke FL (2021). Key challenges for management policies and practices. Towards a human-centred agenda: Human resource management in the BRICS countries in the face of global challenges.

[CR70] Noronha E, D’Cruz P, Banday MUL (2020). Navigating embeddedness: Experiences of Indian IT suppliers and employees in the Netherlands. Journal of Business Ethics.

[CR71] Pandey J, Varkkey B (2020). Impact of religion-based caste system on the dynamics of Indian trade unions: Evidence from two state-owned organizations in North India. Business & Society.

[CR72] Parboteeah KP, Hoegl M, Cullen J (2009). Religious dimensions and work obligation: A country institutional profile model. Human Relations.

[CR73] Parboteeah KP, Hoegl M, Cullen J (2008). Ethics and religion: An empirical test of a multidimensional model. Journal of Business Ethics.

[CR74] Pasquale F (2015). The black box society.

[CR75] Pew Research Center. (2 April, 2015). The future of world religions: Population growth projections, 210–2050. https://www.pewforum.org/2015/04/02/religious-projections-2010-2050/.

[CR76] Porter ME, Kramer MR (2006). The link between competitive advantage and corporate social responsibility. Harvard Business Review.

[CR77] Prasad A, O’Brien LT, Smith Sockbeson CE (2020). Caste at work: Study of factors influencing attitudes toward affirmative action in India. Equality, Diversity and Inclusion.

[CR78] Rogovsky N, Cooke FL (2021). Towards a human-centred agenda: Hu0man resource management in the BRICS countries in the face of global challenges.

[CR79] Ruhe J, Lee M (2008). Teaching ethics in international business courses: The impacts of religions. Journal of Teaching in International Business.

[CR80] Russell S, Norvig P (2016). Artificial intelligence: A modern approach.

[CR81] Schleifer, T. (2020). Google’s former CEO hopes the coronavirus makes people more “grateful” for Big Tech. *recode*, https://www.vox.com/recode/2020/4/14/21221141/coronavirus-eric-schmidt-google-big-tech-grateful.

[CR82] Sen S, Bhattacharya CB (2001). Does doing good always lead to doing better? Consumer reactions to corporate social responsibility. Journal of Marketing Research.

[CR83] Singer AA, van der Ven H (2019). Beyond market, firm, and state: Mapping the ethics of global value chains. Business and Society Review.

[CR84] Singler B (2020). The AI creation meme: A case study of the new visibility of religion in artificial intelligence discourse. Religions.

[CR85] Susskind, D. (2018) Rethinking the capabilities of machines in economics Oxford University Department of Economics Working Paper. University of Oxford, Oxford

[CR86] Syed J, Van Buren HJ (2014). Global business norms and Islamic views of women’s employment. Business Ethics Quarterly.

[CR506] Syed J, Ali HJF (2021). A pyramid of hate perspective on religious bias, discrimination and violence. Journal of Business Ethics.

[CR87] Tambe, P., Hitt, L., Rock, D., & Brynjolfsson, E. (2020). Digital capital and superstar firms. NBER Working Paper 2825. Cambridge, MA: National Bureau of Economic Research

[CR88] Tegmark M (2017). Life 3.0: Being human in the age of artificial intelligence.

[CR89] Thomas, R., & Uminsky, D. (2020) The problem with metrics is a fundamental problem for AI. arXiv preprint arXiv:2002.08512.

[CR90] Tlaiss HA (2015). How Islamic business ethics impact women entrepreneurs: Insights from four Arab Middle Eastern countries. Journal of Business Ethics.

[CR91] Trittin-Ulbrich H, Scherer AG, Munro I, Whelan G (2021). Exploring the dark and unexpected sides of digitalization: Toward a critical agenda. Organization.

[CR92] Vaidyanathan B (2020). How minority religion can shape corporate capitalism: An emergentist account and empirical illustration. Business & Society.

[CR93] Van Buren HJ, Syed J, Mir R (2020). Religion as a macro social force affecting business: Concepts, questions, and future research. Business & Society.

[CR94] van der Ree K, Gironde C, Carbonnier G (2019). Promoting green jobs: Decent work in the transition to low-carbon, green economies. The ILO@ 100.

[CR95] van der Veen RJ, van Parijs P (1986). A capitalist road to communism. Theory and Society.

[CR96] Whelan G (2021). Megacorporation: The infinite times of Alphabet.

[CR97] While A, Eadson W (2021). Zero carbon as economic restructuring: spatial divisions of labour and just transition. New Political Economy.

[CR98] Wilson, H. J., & Daugherty, P. R. (2018). Collaborative intelligence: Humans and AI are joining forces. *Harvard Business Review,* July-August. https://hbr.org/2018/07/collaborative-intelligence-humans-and-ai-are-joining-forces.

[CR99] Winner L (1980). Do artifacts have politics?. Daedalus.

[CR100] Wright CF (2016). Leveraging reputational risk: Sustainable sourcing campaigns for improving labour standards in production networks. Journal of Business Ethics.

[CR102] Xue H, Chan A (2013). The global value chain: Value for whom? The soccer ball industry in China and Pakistan. Critical Asian Studies.

[CR103] Zou, J., & Schiebinger, L. (2018) AI can be sexist and racist—It’s time to make it fair. https://www.nature.com/articles/d41586-018-05707-8. 10.1038/d41586-018-05707-8 30018439

